# Ageing‐Associated Dysregulation of Myogenic Differentiation in Inclusion Body Myositis

**DOI:** 10.1002/jcsm.70301

**Published:** 2026-05-03

**Authors:** Geert M. de Vries, Willem De Ridder, Jonathan Baets

**Affiliations:** ^1^ Translational Neurosciences and Peripheral Neuropathy Group University of Antwerp Antwerp Belgium; ^2^ Laboratory of Neuromuscular Pathology, Institute Born‐Bunge University of Antwerp Antwerp Belgium; ^3^ Department of Neurology, Neuromuscular Reference Centre Antwerp University Hospital Antwerp Belgium

**Keywords:** inclusion body myositis, muscle regeneration, myogenic differentiation, senescence

## Abstract

Skeletal muscle is a postmitotic tissue dependent on a complex and tightly regulated regeneration process involving numerous intracellular and extracellular factors, including myogenic regulatory factors (MRFs), cytokines and myokines. Quiescent satellite cells are activated by physiological stimuli, injury or other traumatic insults for the repair of injuries or growth of the tissue. Activation of satellite cells induces proliferation and expression of MRFs, which in turn activate myogenic differentiation transcription programmes. Transitioning into and committing to terminal differentiation are regulated by myogenin and cell cycle exit markers, notably Rb1 and p21. Differentiation is then complete with the formation of new muscle fibres which incorporate into existing fibres. Upon ageing, the efficiency of differentiation is reduced as a consequence of a loss in the physiological balance between pathways regulating satellite cell quiescence and activation, notably the Notch and Wnt pathways, and increased senescence of the satellite cell pool. Extracellular factors involved in the dysregulation of differentiation upon ageing include low‐grade chronic inflammation and remodelling of the extracellular matrix by fibro‐adipogenic progenitor cells, thereby negatively affecting the differentiation capacity of satellite cells, resulting in either premature differentiation or senescence. These ageing‐associated alterations in muscle homeostasis appear to be amplified in inclusion body myositis (IBM), an idiopathic inflammatory myopathy that almost exclusively manifests in individuals over 45 years of age, making it a prototypical age‐related muscle disease. IBM is characterised by chronic inflammation, progressive muscle degeneration and premature ageing of both muscle tissue and the satellite cell niche. Studied with immunohistochemical techniques and multi‐omics, muscle biopsy tissue demonstrated increased expression of MRFs as well as increased expression of senescence and genomic stress markers. IBM primary myoblasts demonstrated premature ageing and senescence and increased activity of the Wnt pathway, though differentiation into multinucleated myotubes did not show notable aberrations in signalling pathways or differentiation efficiency. In conclusion, ageing and chronic inflammation lead to dysregulation of key pathways that, in turn, alter the capacity of satellite cells to activate and proliferate, leading to prematurely aged satellite cells that still retain their capacity to differentiate into myofibres. Though in IBM there is an increased abundance of active differentiation markers, reflecting a regenerative response to the massive, sustained muscle atrophy, senescence of the satellite cell niche may impair effective regeneration of the lost muscle tissue.

## 1 Introduction

Skeletal muscle is a postmitotic tissue dependent on a complex and tightly regulated regeneration process in response to physiological stimuli such as exercise or growth, as well as injury or traumatic insult, for repair or growth of the tissue [1, 2]. This process starts with the activation of muscle stem cells and ultimately ends with the formation of new muscle fibres which incorporate into existing fibres. Involving multiple cell types, including interstitial and immune cells, and numerous intracellular and extracellular factors, dysregulation can lead to a number of (neuro)muscular diseases that are characterised by progressive wasting of skeletal muscles [3, 4].

The efficiency of muscle regeneration declines with ageing, rendering aged muscle more susceptible to degeneration and incomplete repair [5]. Within this context, progressive muscle wasting is a hallmark of inclusion body myositis (IBM), a late‐onset idiopathic inflammatory myopathy characterised by a remarkably strict age correlation, manifesting almost exclusively after the age of 45 years [6, 7]. IBM presents with slowly progressive muscle atrophy and weakness, initially affecting the deep finger flexors and quadriceps before becoming more generalised [8, 9]. The age‐related occurrence of IBM suggests that disease‐specific mechanisms act upon an age‐associated decline in regenerative resilience, making IBM a compelling model to investigate how ageing‐associated dysregulation of myogenic differentiation contributes to muscle degeneration.

Interestingly, increased numbers of immature regenerating fibres are observed in IBM muscle biopsies, indicating that the muscle retains the capacity to initiate a regenerative response and to activate myogenic progenitors despite the adverse inflammatory and degenerative milieu [10, 11, 12]. However, in the context of continuous muscle loss, this response appears insufficient to counterbalance progressive degeneration. Indeed, accumulating evidence shows dysregulation of both regenerative capacity and differentiation efficiency of myogenic progenitors in IBM muscle [11, 13]. In line with this, therapeutic strategies aimed at enhancing muscle mass or strength have demonstrated only limited benefits, underscoring the complexity of the regenerative process in IBM [14]. On a pathological level, IBM is characterised by the presence of both infiltrating inflammatory cells and rimmed vacuoles, reflecting its dual inflammatory and degenerative phenotype. In addition, features of ER stress, mitochondrial dysfunction and myonuclear abnormalities are observed in diagnostic muscle biopsies [6, 15]. As there is currently no effective treatment, progressive muscle atrophy and subsequent loss of ambulation represent a major unmet medical need [8].

To understand the apparent failure of sustainably improving the muscle mass in IBM and potentially enhance the design of future clinical studies through the identification of novel therapeutic targets or relevant pathways, it is essential to first elucidate the underlying alterations and aberrations in myogenic differentiation in IBM patients. The aim of this review is, firstly, to give a concise overview of the molecular and cellular mechanisms involved in myogenic regeneration, with particular emphasis on the physiological role of key regulatory factors known to be dysregulated in IBM. Secondly, we will examine how ageing and chronic inflammation affect and impair myogenic differentiation, thereby offering deeper insight into the mechanisms driving dysregulated muscle regeneration in IBM.

## 2 Myogenic Differentiation Under Physiological Conditions

### 2.1 Quiescence and Activation of Satellite Cells (SCs)

Maintenance and regeneration of muscle fibres rely primarily on SCs [16]. Located between the basement membrane and plasma membrane of muscle fibres, forming an anatomically distinct niche, SCs are the muscle‐resident stem cells and are activated in response to injury or physiological stimuli, contributing to muscle growth through either fusion with existing myofibres or fusing to form new myofibres [17, 18, 19]. Quiescent SCs are growth‐arrested in G_0_‐phase and show high expression of Pax7 while suppressing the expression of myogenic regulatory factors (MRFs) Myf5 and MyoD [19, 20, 21].

SC quiescence is maintained through a complex interplay between cell and niche and regulated by an array of proteins and signalling pathways, which include, but are not limited to, the crucial mediators Notch, Wnt and p38α/β MAPK [22]. The balance between and activity of these signalling pathways defines and regulates the transitions between quiescence, proliferation, and differentiation of SCs. In quiescent SCs, Wnt and p38α/β MAPK are downregulated or inhibited, while active Notch signalling is required to maintain quiescence. This is effected through its intracellular mediator RBP‐J, which inhibits MyoD expression and promotes Pax7 expression and GSK3β activation, thereby downregulating Wnt signalling activity, promoting noncommitted SCs proliferation and self‐renewal [23, 24]. p38α/β MAPK is generally activated upon injury to muscle tissue and promotes SC proliferation and differentiation in response to sustained muscle damage. In SCs, its inhibition or downregulation of activity can be mediated by the Notch pathway, which drives a return to or maintenance of quiescence [25, 26, 27].

This crucial role of Notch in the regulation of quiescence is emphasised by the observations that enhancing Notch signalling promotes muscle regeneration in aged muscle while its inhibition leads to reduced SC proliferation and self‐renewal [28, 29]. Dysregulation of Notch signalling leads to loss of SC quiescence and excessive myogenic differentiation, mediated by increased Wnt signalling activity as a direct consequence of decreased Notch activity [23, 30, 31, 32].

**FIGURE 1:**
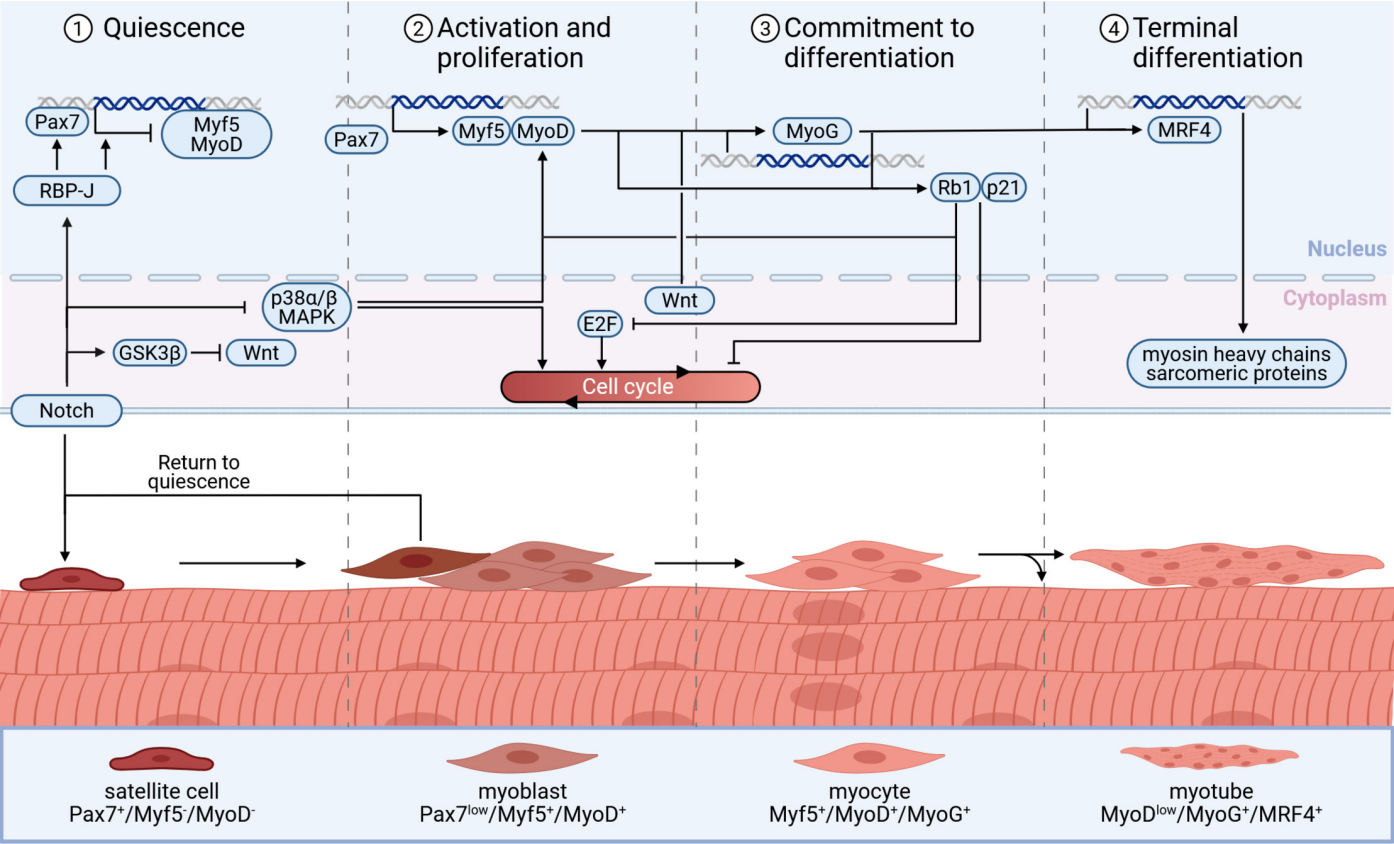
Molecular and cellular mechanism of physiological myogenic differentiation. Physiological myogenic differentiation is initiated by the activation of satellite cells. Quiescent satellite cells express high levels of Pax7, which reduce upon activation and cell cycle entry. As cells start to proliferate, the myogenic differentiation programme is activated by Myf5 and MyoD, with a subpopulation of activated satellite cells reverting to quiescence. Upregulation of myogenin, and subsequent activation of p21, and pRb, commits myoblasts to cell cycle exit and differentiation. Following cell cycle withdrawal, myocytes mature and fuse with existing myofibres or other myocytes, increasing production of myosin heavy chain isoforms and other sarcomeric proteins.

Exit from quiescence and thus activation of SCs initiates a tightly regulated process of proliferation and differentiation [19, 20]. SC activation and cell cycle entry are marked by the downregulation of Pax7 and increased expression of Myf5 and MyoD (Figure 1) [17, 19, 33]. Myf5^+^ SCs give rise to a population of myogenic progenitor cells, while a subpopulation of activated SCs does not express Myf5, renews through asymmetric division and ultimately returns to quiescence [17, 29, 34, 35].

### 2.2 MyoD and Myogenin: Markers of Proliferation and Commitment to Differentiation

MyoD drives the myogenic progenitor phenotype through activation of skeletal muscle‐associated transcription programmes, as evidenced by the transduction of non‐myogenic cell types with MyoD converting these cell lines to myogenic progenitors and giving rise to multinucleated myotubes in vitro [36, 37, 38]. Loss of MyoD function results in a delay in the transition from proliferation to differentiation, as myogenin, an MRF associated with commitment to terminal differentiation, is among MyoD transcription targets [39]. Myostatin, a member of the TGF‐β superfamily of growth‐ and differentiation‐factors, inhibits expression of both MyoD and myogenin, thereby negatively regulating myogenic differentiation [40, 41].

MyoD and myogenin synergistically enhance the expression of Rb1 and cell cycle exit markers p21 and p57, marking the transition from myoblast proliferation to cell cycle exit and commitment to terminal differentiation [2, 34, 42]. In turn, Rb1, suppressing E2F‐dependent genes, acts simultaneously in a positive feedback loop on MyoD, enhancing its expression [42]. While MyoD and myogenin are crucial mediators to commit to the myogenic lineage, Rb1 is an equally important regulator of cell cycle exit and early terminal differentiation, as loss of Rb1 function has been described to result in aberrant differentiation, with Rb1^‐/‐^ myotubes degenerating after 3 days of in vitro culture [37, 43].

Terminal myogenic differentiation is marked by myogenin expression, downregulation of MyoD and absence of Pax7 [2, 33]. Myogenin regulates terminal differentiation through induction of expression of proteins involved in myocyte fusion, notably myomaker/TMEM8C, and sarcomere‐associated proteins [17, 44, 45]. Besides this role, myogenin has been described to be largely dispensable during myogenic differentiation and is eventually downregulated by MRF4, whose expression is induced during the latter stages of differentiation and is described to regulate muscle fibre size [30, 44, 45, 46]. In this stage of terminal differentiation, myocytes fuse with existing myofibres or form new myofibres. During this process, there is an upregulation of sarcomere‐associated proteins, essential for the development of functional contractile muscle tissue. Early in maturation, myosin heavy chain (MYH) type 3 is typically expressed, followed by an isotype switch in later stages for further specialisation into Types I and II muscle fibres [2, 19, 32, 47].

### 2.3 Fibro‐Adipogenic Cells

While myogenic differentiation is predominantly carried out by SCs, interstitial cells of nonmyogenic origin, namely, fibro‐adipogenic progenitors (FAPs), contribute to myogenic differentiation. FAPs are resident mesenchymal cells characterised by the expression of PDGFRa, Sca1 and CD34 surface markers, yet do not express classical SC‐ or myoblast‐associated markers [48, 49]. Though capable of differentiating along either fibrogenic or adipogenic lineage, FAPs contribute to and enhance myogenic differentiation through paracrine signalling, with increasing numbers of FAPs correlated with higher expression of late‐stage differentiation markers [47, 49]. The role of FAPs in enhancing myogenic differentiation is further highlighted through the observation that depletion of FAPs led to reduced muscle mass and myofibre size after several weeks, while in the longer term, a reduction of SCs was observed [48]. In addition, depletion of FAPs resulted in prolonged necrosis and delayed regeneration after acute muscle injury.

Besides their effect on myogenic progenitor cells, FAPs interact with the immune environment through the secretion of IL‐6 and IL‐33, acting on T‐cells, while lymphocyte‐secreted IL‐4 induces fibrogenic differentiation [48]. Absence of IL‐4 signalling, on the other hand, reduces FAP proliferation, induces differentiation into adipocytes and negates its function in clearing cellular debris of damaged myofibres [50].

## 3 Senescence and Chronic Inflammation in the Aged Muscle Niche: Cellular Mechanisms Driving Impaired Regeneration and Reduced Stem Cell Efficiency

This complex and intricate regulatory network underlying physiologic muscle regeneration gradually decreases in efficiency during ageing of an organism, subsequently leading to sarcopenia [20]. Sarcopenia comprises the gradual loss of skeletal muscle mass and a decline in muscle strength as the consequence of complex interactions and intertwining of a range of factors associated with ageing [20]. Though SCs are capable of protecting their quiescence through active inhibition of senescence‐associated pathways, this capacity is increasingly compromised during ageing as major pathways involved in the physiological regeneration of muscle show altered regulation and activity upon ageing, leading to general dysfunction of muscle regeneration [51].The altered balance between the signalling pathways involved in maintaining quiescence or regulating activation and differentiation of SCs leads to downstream consequences, including reduced proliferation and self‐renewal capacity, in turn resulting in gradual exhaustion and senescence of the SC niche [29]. In parallel, the number of myogenic progenitors primed for activation and proliferation is increased, with concomitant increases in myogenin expression, indicating that activation of SCs and commitment to differentiation are highly dysregulated [51, 52, 53].

## 4 Intrinsic Changes in SCs as Consequence of Ageing: Alterations in Signalling Pathways Lead to Loss of Quiescence and Premature Differentiation

The dysregulation of myogenic differentiation with ageing and the subsequent sarcopenia is the consequence of broadly two processes: the loss of SC quiescence regulation and the increased expression of senescence‐associated markers that accompany and partially underlie sarcopenia [20]. Senescence is a state of irreversible cell‐cycle arrest and phenotypic alterations induced by a number of stressors, such as unresolved DNA damage or telomeric attrition, and also inflammation, excessive ROS production and loss of proteostasis [54, 55, 56]. These stressors contribute to ageing through different mechanisms. For example, compromised DNA integrity and unresolved DNA damage can lead to cell cycle arrest and senescence [56, 57]. In addition, mitochondrial dysfunction impacts global energy homeostasis and leads to excessive ROS generation [56]. Loss of proteostasis is a general ageing‐related mechanism prompted in part by dysregulation of key metabolic pathways such as the mTOR pathway, a pathway that is also involved in myogenic differentiation [58, 59, 60]. On a cellular level, senescence leads to a range of changes in cellular processes, including decreased proliferation and differentiation, as well as further impairment of metabolic homeostasis and altered expression of markers of DNA damage repair and cell cycle [20, 54, 56, 61].

**FIGURE 2:**
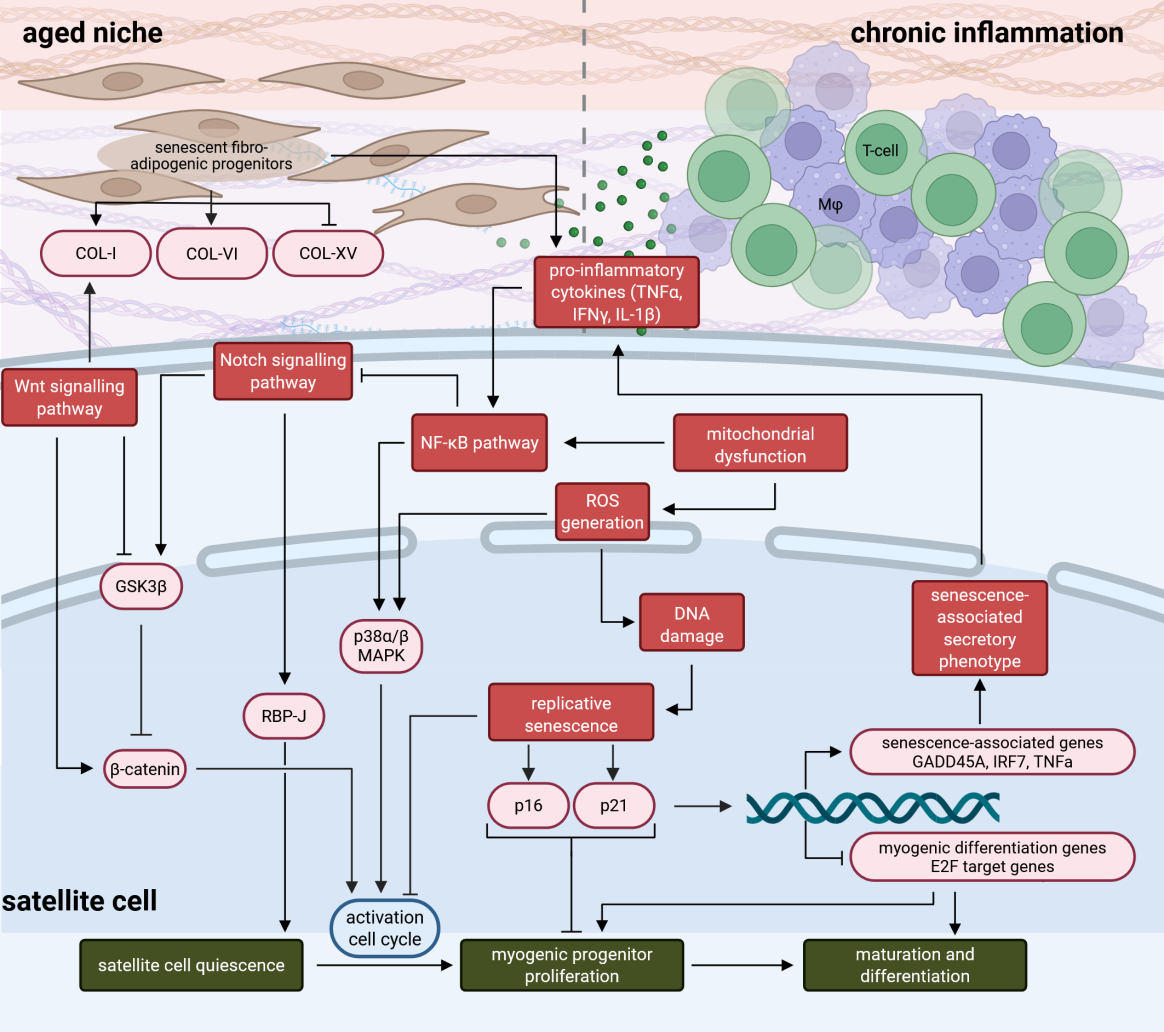
Intrinsic changes in satellite cells and senescence and inflammation in the myogenic niche lead to alterations in key processes in myogenic differentiation. Fibro‐adipogenic progenitors (FAPs) and immune cells act as extrinsic regulators that negatively affect the myogenic niche through altered collagen deposition and secretion of pro‐inflammatory cytokines. In turn, these alterations influence processes in the satellite cell through activation of the NF‐κB pathway and dysregulation of the Notch and Wnt signalling pathways. Downstream effects include mitochondrial dysfunction, cellular senescence and altered expression of genes involved in myogenic proliferation and differentiation. Ultimately, these age‐ and inflammation‐associated changes disrupt the entire myogenic differentiation cascade.

Major signalling pathways involved in these alterations and the subsequent dysfunction of muscle tissue in aged individuals are Notch and Wnt, both involved in the regulation of regeneration. In aged muscle, Wnt signalling shows increased activity as evidenced by decreased active GSK3β and translocation of active β‐catenin to the nucleus, while simultaneously, there is a decrease in Notch expression and subsequent activity of the signalling pathway (Figure 2) [23, 28, 62]. This increase in Wnt signalling in early activated SCs shifts the balance with Notch to reduced proliferation and premature differentiation, thereby impairing effective regeneration [63]. In addition, effective regeneration is also impaired as a consequence of elevated Wnt signalling due to the pro‐fibrotic effects, leading to increased collagen I deposition and reduced progenitor proliferation [16].

In addition, p38α/β MAPK and p21 are both increased in activity upon ageing and associated with senescence [23, 56, 64, 65]. Under physiological conditions, p38α/β MAPK is activated by cellular stressors, including ROS production and inflammatory cytokines, and plays a role in proliferation and differentiation of SCs, while its inhibition maintains SC quiescence [26]. In aged muscle, however, p38α/β MAPK is increasingly active, thereby promoting premature cell cycle entry and differentiation, in part, in response to the inflammageing‐associated muscle environment [64, 66]. In turn, this activation in aged muscle cells leads to diminished self‐renewal capacity as well as increased entry into a senescent state [25, 32]. Pharmacological inhibition of p38, however, leads to rescue of impaired self‐renewal of aged SCs [24, 28].

As a hallmark marker of senescence, p21 is activated during physiological differentiation by MyoD and leads to cell cycle exit and commitment to the differentiation programme [19, 34]. In aged muscle tissue, p21 expression is increased, which, together with activated p53 and pRb, reflects increased cell cycle arrest [64, 67]. This increased expression of p21, however, has detrimental effects on muscle tissue and resident cells. Overexpression of p21 in mice led to increased expression of senescence‐associated secretory phenotype (SASP)‐related factors, a collection of pro‐inflammatory cytokines, chemokines and growth factors. In turn, this senescent secretory phenotype elicits increased expression of stress and inflammation markers GADD45A, IRF7 and TNFα and promotion of tissue fibrosis [68]. In addition, inflammation and impairment of muscle function as consequence of p21‐induced senescence were observed in murine myofibres [69]. On a transcriptional level, p21 overexpression induces increased Rb1 hypophosphorylation, which in turn leads to downregulation of E2F‐dependent genes, thereby inhibiting cell cycle progression [42, 70]. Geriatric SCs transplanted into a young host muscle retained activation of the p16–Rb1 axis and persistent repression of E2F‐dependent transcription, underscoring that age‐associated senescence is maintained as a cell‐intrinsic process [51].

## 5 Extrinsic Changes: Senescence of Niche and Chronic Inflammation

In the muscle niche, senescence has far‐reaching consequences for both the extracellular compartment and the regenerative capacity. The increased expression of p21, reflecting senescence, severely reduces the proliferative capacity of SCs and interstitial resident cells. Accumulation of senescent cells in the muscle niche is associated with degenerative phenotypes, remodelling of the extracellular matrix (ECM) and ageing of the surrounding tissue through the adoption of the SASP, characterised by the secretion of pro‐inflammatory cytokines, chemokines and growth factors [54, 71, 72].

### 5.1 Senescent FAPs

While senescent SCs are still capable of regenerating myofibres, though to a reduced extent, senescence in FAPs alters the myogenic potential and the ECM more dramatically. Aged FAPs display a higher propensity to a pro‐fibrotic phenotype, shifting from collagen XV to collagen I deposition, leading to fibrosis and increased stiffness of the ECM [73, 74]. In addition, senescent FAPs compromise muscle function through the secretion of SASP‐associated cytokines, thereby promoting low‐grade chronic inflammation. In short, senescent FAPS compromise muscle function and negatively modulate the tissue environment, thereby compromising SC proliferation and efficient differentiation [47].

### 5.2 Chronic Inflammation

Although a certain degree of inflammation is a physiological and even required element of efficient myogenic differentiation, sustained or chronic inflammation has far‐reaching consequences for the integrity and function of muscle tissue [75, 76]. With ageing comes a decline in immune function, thereby leading to low‐grade chronic inflammation or unresolved immune responses, a process termed inflammageing [55, 77]. Chronic stimulation of the NF‐κB pathway by pro‐inflammatory cytokines IL‐1β, TNFα or IFNγ negatively impacts myogenic differentiation. Activated by the NF‐κB pathway, the p38α/β MAPK pathway interferes with maintenance of SC quiescence, leading to aberrant activation and proliferation [26, 78, 79]. Proliferation of myogenic progenitors is affected through the inhibition of several crucial factors, notably Notch, inhibited by TNFα and thereby negatively impacting proliferation, and downregulation of MyoD, leading to impaired transition from proliferation to differentiation [22, 80, 81, 82, 83]. Terminal differentiation, in turn, is affected by chronic activation of the NF‐κB pathway through its inhibitory effects on the expression of myogenin and MYH isoforms [40, 78, 83, 84]. Prolonged exposure to IFNγ in mice led to general decreases in myogenic differentiation efficiency, as evidenced by decreased expression of MRFs and inhibition of myocyte fusion, reduced SC activation and proliferation and increased collagen deposition [76]. Moreover, IFNγ, prominently expressed in IBM muscle, can induce mitochondrial dysfunction and p16/p21‐driven senescence in myogenic cells, thereby directly coupling chronic inflammation to impaired regenerative capacity [76, 85].

Through the chronically activated NF‐κB pathway, pro‐inflammatory cytokines secreted by immune cells or tissue‐resident cells elicit the activation of several pathways involved in regulation of quiescence and activation of SCs. The activated myogenic progenitors, in turn, are negatively affected by the chronic inflammatory environment as these cells display reduced proliferative and differentiation capacity. Eventually, this impaired ability to repair damaged skeletal muscle contributes to overall muscle wasting [83].

## 6 Myogenic Differentiation in IBM: Increased Proliferation and Differentiation Markers and Upregulation of Senescent Signatures

The interplay between chronic inflammation and premature ageing underlies IBM pathophysiology with far‐reaching consequences for the niche and efficacy of myogenic differentiation. As IBM is characterised by concomitant inflammation and degeneration as a consequence of this interplay, research mainly focused on the immunological component and the general loss of proteostasis, a hallmark of ageing and consequence of chronic inflammation [56, 86]. These pathomechanisms and general alterations underlying IBM have predominantly been studied using patient muscle biopsy tissue, with a substantial number of studies contributing to an ever‐increasing understanding of IBM pathology and the interactions between cellular and molecular players [15]. While previously the alterations on tissue level, with notably reduced myofibre cross‐section area and loss of Type II fibres, have been described, the age‐related aspects and the consequences of premature ageing on myogenic differentiation in IBM remain understudied [86, 87].

### 6.1 Human Muscle Biopsy Studies

**FIGURE 3:**
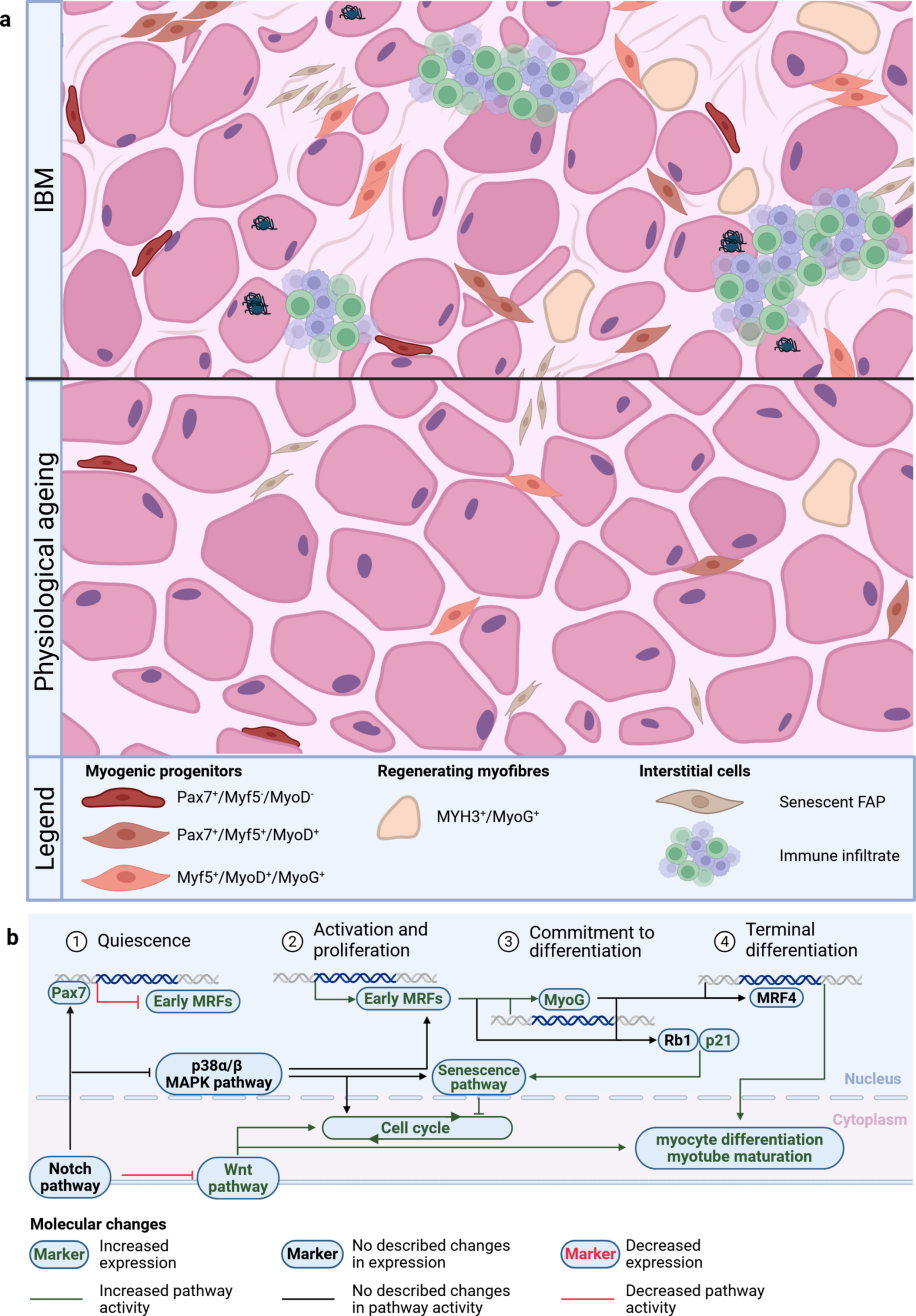
Strong regenerative response in inclusion body myositis as result of increased activation and proliferation of myogenic progenitors. (A) Cross‐sectional representation of muscle tissue in inclusion body myositis (IBM) compared with physiologically aged muscle tissue. In physiologically aged muscle, only limited activation of satellite cells and subsequent differentiation occurs. In contrast, IBM muscle shows a strong myogenic regenerative response to the sustained damage and degeneration of myofibres, reflected by the increased presence of active and proliferating myogenic progenitors in different stages of commitment and differentiation. This increased presence of myogenic progenitors in turn contributes to increased differentiation activity and a higher number of maturing myofibres. (B) Dysregulation of the myogenic differentiation pathway at the molecular level is reflected by increased expression of several markers associated with proliferation and differentiation stages of myogenic progenitors. Disbalance of Notch and Wnt signalling activity contributes to increased cell cycle activity, whereas increased senescence inhibits proliferation of a subset of myogenic progenitors. Several key regulators of satellite cell activation, proliferation and differentiation appear to show no marked differences in expression or activity.

Studies focusing on myogenic regeneration in IBM demonstrated in patient muscle tissue an increased abundance of Pax7^+^ SCs and myogenic cells expressing differentiation markers MyoD and myogenin compared to healthy controls [11, 12]. These findings are complemented by a recent study detecting reduced myostatin serum levels and gene expression in IBM patient muscle biopsies while showing increased myogenin and Myf5 expression (Figure 3A,B) [88]. Activation and extensive proliferation of SCs and myogenic cells, potentially reflecting reduced maintenance of quiescence, were also observed through the increase of Ki67‐positive nuclei in muscle biopsies [70]. In addition to nuclear markers, upregulation of CD56, developmental myosin heavy chain isoform MYH3 and vimentin was found during myogenic differentiation and their expression increased in IBM muscle tissue compared to controls [89].

### 6.2 Myopathological Studies

In amyloid‐beta overexpressing murine myotubes, modelling a hypothesised primary degenerative pathology driver, an increase in Ki67‐ and PCNA‐positive nuclei was found [70]. In one in vivo mouse model with xenografted IBM patient‐derived muscle tissue, increased cross‐sectional area was observed, while no differences were found in myofibre maturation [90]. However, one study found increased DNA damage in myonuclei and impaired DNA damage repair in IBM muscle tissue, which can contribute to accelerated ageing and impaired regeneration [91]. Collectively, these findings indicate that IBM muscle exhibits increased activation of SCs and myogenic regenerative activity in response to the sustained chronic myodegeneration in the aged environment characterising IBM, while terminal myofibre maturation appears largely preserved.

### 6.3 Findings From Transcriptomics and Proteomics Studies

A relatively small but growing number of studies has also employed proteomics or transcriptomics on IBM muscle biopsy tissue. In studies employing transcriptomics, common findings include a senescent signature, increased interferon Type II activity and alterations in myogenic differentiation and ECM components. In particular, upregulation of general senescence markers CDKN1A (p21) and CDKN2A (p16^INK4A^) and GADD45A, as a marker of genomic stress, was detected [74, 92, 93, 94, 95]. In line with the findings from the immunostainings on muscle biopsy tissue, transcriptomic studies found increased expression of markers of different myogenic differentiation stages (myogenin, TMEM8C/Myomaker and neonatal MYH3), while also a shift in collagen fibre type (upregulation of Types VI and I and downregulation of type XV) was observed [89, 92, 94, 96, 97].

While with bulk‐RNA sequencing, discrimination between resident cell types (myofibres, FAPs, SCs, immune cells, etc.) is not evident, two studies employed single‐nuclei RNA sequencing, detecting both a selective loss of fibre Type II myonuclei, while one study found FAPs to be the predominant cell type, demonstrating a clear senescent phenotype [74, 93]. In addition, a shift in collagen expression from Type XV to Type I, observed with single‐nuclei RNA sequencing, resulted in a decreased survival of primary human muscle cells, reflecting that senescent FAPs negatively modulate the regeneration niche by altered collagen deposition [74].

Proteomics studies have not consistently detected proteins typically associated with senescence directly. In line with the transcriptomics studies, upregulation of collagen Types VI and I and increased expression of MYH3 are observed, while in addition, the downregulation of fast‐twitch MYHs 2 and 4 is observed [10, 98, 99, 100, 101]. Going beyond the differential expression of proteins between IBM patients and controls, one study explored upstream regulators, proteins that might drive these observed changes and reveal deeper layers of proteome dysregulation [10]. This analysis identified altered regulation of key myogenic differentiation proteins, including KDM5A and Rb1, alongside decreased activity of critical transcription factors MEF2C and MyoD, suggesting that both SC activation and differentiation programmes are modified in the aged IBM muscle environment, potentially contributing to impaired regenerative capacity despite active myogenic signalling.

### 6.4 Findings From Cell‐Based Studies

Complementing the findings of the muscle biopsy immunohistochemical stainings, in vitro studies using primary cells from IBM patients show more dynamically the alterations and impairments of myogenic differentiation in IBM patients' muscle. While the muscle sections demonstrate an increased regenerative response to chronic muscle injury and inflammation, primary cells isolated from IBM patient muscle tissue nuance that picture.

Only one study has so far described the characteristics of primary myoblasts isolated from patient muscle biopsies. These myoblasts were found to have a significantly reduced proliferative and clonogenic capacity and significant telomere shortening compared to primary myoblasts isolated from age‐matched healthy controls, indicating premature ageing and senescence of myogenic progenitors in IBM patients and highlighting their difficulties in in vitro culture conditions. Despite the senescent phenotype, these primary myoblasts differentiated into multinucleated myotubes, demonstrating a similar fusion index compared to healthy controls. Though no apparent aberrant activation or dysregulation of skeletal muscle differentiation signalling pathways was observed, increased activity of the Wnt signalling pathway was observed [13].

In a study by the same lab, mesoangioblasts were isolated from IBM patient muscle tissue [38]. Mesoangioblasts have been described to differentiate along the myogenic lineage in co‐culture conditions with myoblasts or in vivo upon injury [102, 103, 104]. However, IBM mesoangioblasts failed to express the classical markers MyoD, myogenin or MYH, compared to dermatomyositis controls, and did not differentiate into myotubes in vitro. Activation of the p38 MAPK pathway, involved in differentiation but also senescence, did not show aberrations, while no reduced or impaired proliferation capacity was reported [38].

Besides primary myoblasts and mesoangioblasts, patient‐derived fibroblasts have been used to generate iPSCs in one study. Though not describing the fusion index, the measure to express differentiation, bulk RNAseq data on IBM iPSC‐derived myotubes shows significant increases compared to control iPSCs in the classical differentiation markers MyoD, Myogenin, MYH3 and a range of sarcomeric proteins [105].

## 7 Discussion

IBM is a strictly age‐related inflammatory myopathy that almost exclusively manifests in individuals over 45 years of age, emerging within an aged muscle environment [6]. Ageing is associated with a progressive decline in efficiency and scale of myogenic differentiation, contributing to reduced regenerative capacity and sarcopenia [20]. However, beyond this gradual functional decline, aged muscle represents a state of diminished biological resilience characterised by cumulative mitochondrial dysfunction, impaired proteostasis, chronic low‐grade inflammation and SC senescence [5, 72]. Together, these processes destabilise the regenerative system and lower the threshold at which degeneration outweighs repair.

The age‐associated mitochondrial dysfunction, proteostatic failure, and disruption of key regulatory pathways governing SC quiescence and differentiation create a substrate of vulnerability upon which disease‐specific factors, including IFNγ signalling and inflammatory stress, may act [77, 106]. This environment induces cellular senescence of the myogenic niche, leading to reduced sensitivity of SCs to mitogenic cues and activation signals, while FAPs negatively modulate the niche, thereby contributing to impaired activation, proliferation and differentiation of myogenic progenitors [74, 78]. The strict age distribution supports the notion that IBM does not arise in young, resilient muscle but rather emerges within this biologically permissive aged environment. In this context, IBM may reflect an amplification of age‐related decline in regenerative resilience, ultimately resulting in progressive dysregulation and failure of effective myogenic differentiation.

Characterised by severe myofibre degeneration and chronic inflammation, IBM displays severe muscular atrophy, with findings from studies pointing towards premature ageing as the consequence of primary events that currently remain unclear [10, 13, 15, 74]. As muscle tissue is lost to the degenerative and inflammatory processes, a regenerative response is mounted to compensate. Considering the effects of senescence and chronic inflammation on myogenic differentiation, this regenerative response would be limited in scale and less efficient as the activation and proliferation of SCs is decreased, predominantly through IFNγ and p21 signalling [67, 76]. However, reviewing the current literature reveals several seemingly paradoxical observations in the IBM literature on myogenic differentiation.

On one side of the paradox are the observations of increased presence of cell cycle and proliferation markers in IBM patient muscle tissue and the increased expression of myogenic differentiation markers. On the other side are the downstream effects of chronic inflammation and increased senescence in SCs and their myogenic progeny, as well as FAPs [11, 12, 70, 74, 92]. A comprehensive explanation for this conundrum, why there is increased regeneration occurring when there should be a severely limited response mounted in terms of cellular proliferation and differentiation potential, is not straightforward, as the exact factors and their interplay underlying the dysregulation of myogenic differentiation remain poorly described, largely due to a lack of experimental data and in‐depth studies. Despite this, several clues are discernible to the mechanisms and interactions at play.

Reflecting on one hallmark of IBM, the marked chronic inflammation, the prolonged exposure to pro‐inflammatory cytokines and the ensuing activation of the NF‐κB pathway, in particular through IFNγ signalling, which is characteristic in IBM, have detrimental effects on proliferation and the myogenic differentiation cascade [76, 83]. Chronic IFNγ exposure may accelerate age‐associated loss of regenerative resilience by promoting mitochondrial dysfunction, cellular senescence and activation of atrophy programmes, which are markedly present in IBM patient muscle tissue, thereby reinforcing the permissive aged environment in which dysregulated differentiation emerges [74, 76, 85]. The strong IFNγ signature observed in IBM may therefore represent more than an epiphenomenon of immune activation. However, in other myositis subtypes, notably dermatomyositis, immune‐mediated necrotising myositis, and polymyositis, increased regeneration was observed [10, 11]. Moreover, one additional study focusing on patients with polymyositis with mitochondrial pathology, a myositis subtype proposed to be a precursor stage of IBM, found a positive correlation between activation of the interferon pathway with Pax7 and markers of differentiation (myogenin, MYH3 and MYH8) [95]. The increased regeneration under inflammatory conditions may suggest that this inhibitory effect is of less importance than may be previously assumed. However, it is important to note that other factors, including disease duration and, by proxy, the exposure time to chronic inflammation, may be of relevance.

Prolonged exposure to pro‐inflammatory cytokines can lead to the adoption of a senescent phenotype, which in turn can contribute to inflammation through the secretion of cytokines [74, 76, 107]. While prolonged exposure to external stressors, in the form of pro‐inflammatory cytokines, is one of the ways a cell can senesce, one of the other major causes is replicative senescence. This can arise as a consequence of repeated mitosis or cell cycling, leading to telomeric attrition and withdrawal from the cell cycle [11, 107, 108].

In IBM, while there is reduced proliferation and clonogenicity in vitro, which is in line with the expected effects of senescence, ex vivo, there is increased cell cycling and proliferation of myogenic progenitors [11, 13]. The increased cell cycling may in part be attributable to a subpopulation of SCs that has not (yet) adopted a senescence phenotype and retains their activation potential, which is reflected by the reduced clonogenicity observed.

Interestingly, in vitro cell culture of IBM patient‐derived myoblasts also demonstrated increased activation of the Wnt signalling pathway [13]. With the Wnt pathway regulating Notch and their balance governing key functions in muscle biology, namely, the regulation of SC quiescence, activation and differentiation, dysregulation would have detrimental effects [19]. While p38α/β MAPK has additional roles in these processes, dysregulation of the Notch and Wnt pathways seems to take on an essential role in the dysregulation of SC activation and myogenic differentiation [23, 82].

As Notch plays a key role in maintaining quiescence and returning to quiescence of SCs during asymmetric cell division, loss or suppression of this signalling pathway through Wnt overactivity would eventually lead to exhaustion of the SC pool. Interestingly, the findings in IBM myoblasts and the effects of Wnt signalling overactivity are supported by another study that found that stimulation of Wnt signalling resulted in increased expression of myogenin, MYH3, and myomaker, demonstrating promotion of commitment to terminal myogenic differentiation [109]. The increased expression of these markers in vitro in transdifferentiated myoblast‐like cells upon increased Wnt signalling echoes the findings of the IBM transcriptomics studies, providing further support for the involvement of overactive Wnt signalling (or aberrant Notch signalling) in IBM muscle tissue.

A recent finding adds to these observations, namely, the identification of KDM5A overactivity in the IBM muscle proteome as a potential upstream driver of pathology [10]. Though more research into the role and exact mechanism through which KDM5A is involved is required, its link with both the Notch and Wnt pathways is striking in light of their function in healthy myogenic differentiation and their dysregulation in IBM muscle tissue. In particular, KDM5A is linked to decreased Notch activity through repression of Notch target genes upon formation of a repressor complex with RBP‐J, thereby enabling increased Wnt signalling activity [110]. In itself, KDM5A is involved in transcriptional regulation, predominantly transcriptional repression, of mitochondrial proteins, while in conjunction with Rb1, it can regulate cell cycle and differentiation [37]. These processes show aberrations upon ageing and can contribute to the (accelerated) ageing of cells and tissues.

**FIGURE 4:**
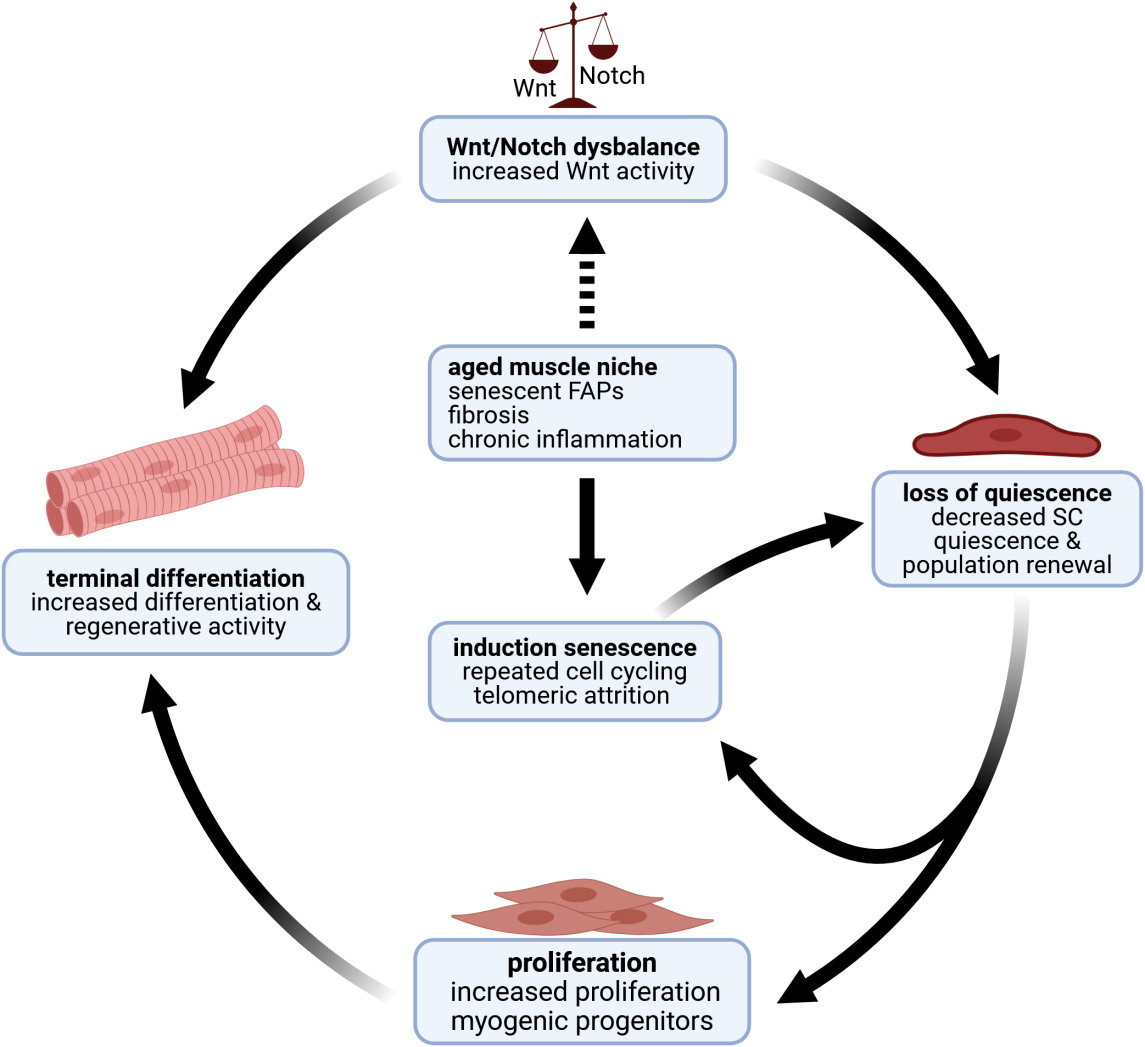
Hypothesised mechanism underlying dysregulation of myogenic differentiation in inclusion body myositis. Based on current literature, it can be hypothesised that dysregulation of the Notch and Wnt signalling pathways may play a central role in the disturbed myogenic differentiation observed in IBM. This dysregulation may impair maintenance of satellite cell quiescence and promote excessive proliferation of myogenic progenitors. Ultimately, the increased activity of Wnt signalling may drive these myogenic progenitors towards premature differentiation, potentially explaining the strong regenerative response observed in IBM. In addition, the increased proliferation of myogenic progenitors may contribute to exhaustion of the satellite cell pool, leading to telomeric attrition as consequence of repeated cycling and cellular senescence.

As differentiation pathways appear largely intact in IBM, dysregulation of the cell cycle emerges as a central mechanism. This may result from increased Wnt pathway activity, which suppresses Notch signalling and disrupts maintenance of quiescence, and from KDM5A overactivity, which promotes cell cycle progression. The age‐related imbalance between these pathways promotes premature SC activation, replicative stress and progressive exhaustion of the SC niche, ultimately reducing the regenerative reserve and compromising effective muscle repair. With a larger number of active myogenic progenitors, the increased activity of the Wnt pathway may then induce (premature) differentiation, supporting the observations of increased expression of MRFs and differentiation markers (Figure 4). This, however, is a hypothesis that needs to be tested in appropriate and optimal models and over a time course to study in‐depth the progression of the regenerative response.

### 7.1 Current Limitations

So far, no time‐series data are available in IBM studies. Most, if not all, studies discussed here have focused on general pathological patterns or senescence, dissecting the transcriptional and signalling pathways found to be dysregulated in more detail. However, while providing some insights into myogenic differentiation, these studies remain a cross‐section of the diseased muscle. This study design leaves the questions of disease progression and, most crucially, the balance between regeneration and degeneration of myofibres over time, unfortunately, unanswered. Studies addressing these questions could provide novel insights into the causes of premature ageing in IBM muscle and the downstream loss of proteostasis, inflammageing and the increased senescence of SCs and FAPs as observed. In addition, it should be noted that the literature on myogenic differentiation in IBM, especially with regard to cell‐based studies, is sparse, with the few studies present in the current literature reviewed here. Though not a limitation in the classical sense, a thorough and detailed understanding of the in vitro differentiation dynamics of IBM patient‐derived myoblasts is currently lacking. While isolation of primary myoblasts has been shown to be a challenging exercise, predominantly limited by the increased tendency of IBM patient‐derived myoblasts to senesce, such studies could provide valuable insights into the currently understudied regeneration dynamics and their shortcomings in IBM [13].

### 7.2 Future Directions

The implications of the aged environment on myogenic differentiation are hard to overstate, with ageing‐related research emphasising the detrimental effects of ageing on the regeneration of tissue [5, 72]. Previous pharmacological interventions designed to sustainably improve muscle mass and function have so far been mostly focused on TGF‐β‐related pathways and targets [14]. However, all these clinical trials failed to reach their primary endpoints, with muscle tissue not sustainably restored. Curiously, as the focus of several clinical trials, the evidence for an essential role of the TGF‐β pathway in myogenic differentiation in IBM is sparse.

The central role of the Notch and Wnt pathway raised in this review highlights the need to explore new therapeutic targets and avenues. In this context, KDM5A has previously been proposed as a potential novel therapeutic target in IBM, given its involvement in modulating Notch and Wnt signaling and its direct role in myogenic differentiation [10]. In vivo modulation of the Notch and Wnt pathways has previously shown beneficial effects on ageing and differentiation, supporting the rationale for further investigation in IBM [82, 111]. Wnt inhibitors, widely used in oncology, may be repurposed for IBM, although their safety and efficacy in this context require thorough evaluation. Another potential therapeutic candidate is sirolimus (also known as rapamycin), currently under investigation in clinical trials for IBM. However, the role of the mTOR pathway in IBM, and its potential to address age‐related dysfunction in myogenic differentiation, remains unclear [112, 113].

## 8 Conclusions

To summarise, myogenic differentiation in IBM is increased in activity and does not show evident intrinsic defects, despite the aged and chronically inflamed microenvironment and the presence of senescent, prematurely aged FAPs and SCs. This sustained differentiation activity in IBM is suggested to be the consequence of age‐related dysregulation of Notch and Wnt signalling, which, together with pro‐inflammatory processes, may drive premature ageing of SCs. Although direct mechanistic evidence remains limited, these insights into the dysregulation of pathways and factors governing myogenic differentiation in IBM open new avenues for research and highlight potential novel therapeutic targets aimed at more effectively counteracting muscle wasting in this age‐related disease.

## Conflicts of Interest

The authors declare no conflicts of interest.
